# A hybrid genome assembly of the endangered aye-aye (*Daubentonia madagascariensis*)

**DOI:** 10.1093/g3journal/jkae185

**Published:** 2024-08-07

**Authors:** Cyril J Versoza, Susanne P Pfeifer

**Affiliations:** Center for Evolution and Medicine, School of Life Sciences, Arizona State University, Tempe, AZ 85281, USA; Center for Evolution and Medicine, School of Life Sciences, Arizona State University, Tempe, AZ 85281, USA

**Keywords:** aye-aye, *Daubentonia madagascariensis*, Daubentoniidae, strepsirrhine, primate, hybrid assembly

## Abstract

The aye-aye (*Daubentonia madagascariensis*) is the only extant member of the Daubentoniidae primate family. Although several reference genomes exist for this endangered strepsirrhine primate, the predominant usage of short-read sequencing has resulted in limited assembly contiguity and completeness, and no protein-coding gene annotations have yet been released. Here, we present a novel, fully annotated, chromosome-level hybrid de novo assembly for the species based on a combination of Oxford Nanopore Technologies long reads and Illumina short reads and scaffolded using genome-wide chromatin interaction data—a community resource that will improve future conservation efforts as well as primate comparative analyses.

## Teaser

The aye-aye (*Daubentonia madagascariensis*) is the only extant member of the Daubentoniidae primate family. Versoza and Pfeifer present a novel, fully annotated, chromosome-level hybrid de novo assembly for the species – a community resource that will improve future conservation efforts as well as primate comparative analyses.

## Introduction

The aye-aye (*Daubentonia madagascariensis*), a strepsirrhine endemic to Madagascar, is the only extant member of the Daubentoniidae primate family. Despite exhibiting the widest geographical distribution within the Lemuroidea superfamily (Sterling 1994) and few natural predators (Richard and Dewar 1991), rapid habitat destruction (Suzzi-Simmons 2023) has resulted in a sharp population decline of ≥50% since the 1980s (Louis *et al*. 2020). Exploitation through human hunting activities further threatens the survival of the species, targeting aye-ayes not only as a source of food and to limit the loss of agricultural crops that they consume but also due to a regional Malagasy cultural belief that aye-ayes are an omen of misfortune, illness, and death (Andriamasimanana 1994). Due to these ongoing vertiginous trends, a further >50% population decline is expected over the next 3 generations (i.e. within 10–24 years), making aye-ayes one of the 25 world’s most endangered primate species, according to the International Union for Conservation of Nature and Natural Resources Species Survival Commission Primate Specialist Group (Schwitzer *et al*. 2013; Louis *et al*. 2020; and see the discussion in Gross 2017).

Due to the species' nocturnal and solitary behavior as well as extensive individual territories (ranging up to 20 and 80 acres for females and males, respectively), direct observation and invasive sampling of individuals are challenging for population monitoring and conservation. Circumventing this difficulty, recent work by Aylward *et al*. (2018) demonstrated the usage of mitochondrial genomes isolated from environmental DNA obtained from saliva deposited at feeding traces for the genetic characterization of aye-aye populations. Importantly, such target-capture strategies strongly rely on the quality of the genomic resources available for the species of interest, necessary for the design of baits needed to distinguish endogenous (i.e. aye-aye) DNA from exogenous DNA (originating, for example, from plant or microbial sources).

The first whole-genome assembly for the species, DauMad_1.0 (NCBI GenBank accession number: GCA_000241425.1), used by Aylward and colleagues, was published in 2012 (Perry *et al*. 2012). Based on medium-coverage (∼20×) 100 bp paired-end Illumina Genome Analyzer IIx sequencing data, this unfinished draft genome consists of 3,231,305 scaffolds (scaffold N50: 3.7 kb; scaffold L50: 193,839) spanning a genome size of 2.9 Gb (Table 1). Nearly a decade later, the Zoonomia Consortium (2020) published a second version, DauMad_v1_BIUU (GCA_004027145.1), built from high-coverage (∼75×) 250 bp paired-end Illumina HiSeq2500 sequencing data, that exhibits an order of magnitude fewer scaffolds (number of scaffolds: 342,451; scaffold N50: 379.9 kb; scaffold L50: 1,894) and a total genome size of 2.5 Gb. Additionally, based on short-insert size 150 bp paired-end Illumina HiSeq X data combined with genome-wide chromatin interaction data (i.e. Hi-C reads), the DNA Zoo team (https://www.dnazoo.org/) generated a highly contiguous chromosome-length assembly of 2.4 Gb length (number of scaffolds: 103,752; scaffold N50: 211.5 Mb; scaffold L50: 5). Although an improvement over the previous versions, the predominant usage of short-read data continued to render parts of the genome inaccessible due to their high repeat content (see the discussion in Logsdon *et al*. 2020). To overcome issues of incompleteness and fragmentation, ∼60× coverage single-molecule PacBio RSII long reads have recently been used to generate the first long-read assembly for the species, ASM2378347v1 (accession number: GCA_023783475; Shao *et al*. 2023). However, unlike earlier versions, this latest assembly is at the contig level (number of contigs: 2,701; contig N50: 28 Mb; contig L50: 26), spanning 2.41 Gb out of an estimated 2.59 Gb. Notably, no protein-coding gene annotations were released for any previous genome assembly.

**Table 1.** Contiguity and completeness of aye-aye genome assemblies.

		DauMad_1.0	DauMad_v1_BIUU	DNA Zoo	ASM2378347v1	DMad_hybrid (this study)
	# scaffolds	3,231,305	342,451	103,752	2,701	696
	N50 (scaffold)	193 kb	379 kb	211 Mb	27 Mb	215 Mb
	# contigs	3,527,892	344,978	122,376	2,701	930
	N50 (contig)	209 kb	298 kb	215 kb	27 Mb	80 Mb
	L50	193,839	1,894	5	26	5
	Total length (in bp)	2,855,365,987	2,498,418,007	2,433,754,680	2,412,003,188	2,440,096,787
	% gaps	0.26%	0.01%	0.26%	0.00%	0.01%
	# annotated genes	–	—	—	—	18,858
Complete BUSCOs	Eukaryota (%)	63 (24.71%)	247 (98.86%)	253 (99.22%)	252 (98.82%)	254 (99.61%)*^a^*
	Mammalia (%)	1,225 (13.28%)	8,504 (92.17%)	8,883 (96.28%)	8,881 (96.26%)	9,220 (99.93%)*^a^*
	Primates (%)	1,786 (12.96%)	12,445 (90.31%)	13,008 (94.40%)	13,016 (94.46%)	13,668 (99.19%)*^a^*

^*a*^99.22%, 97.72%, and 96.49% at the transcript level for eukaryota, mammalia, and primates.

Leveraging the strengths of several orthologous genomic technologies, we here present a novel, fully annotated, chromosome-level hybrid de novo assembly of the endangered aye-aye (DMad_hybrid) that improves both the contiguity and completeness of the genome for future conservation studies and primate comparative analyses.

## Materials and methods

### Animal subjects

This study was approved by the Duke Lemur Center's Research Committee (protocol BS-3-22-6) and Duke University’s IACUC (protocol A216-20-11). The study was performed in compliance with all regulations regarding the care and use of captive primates, including the US National Research Council's Guide for the Care and Use of Laboratory Animals and the US Public Health Service's Policy on Human Care and Use of Laboratory Animals.

### Sample collection, preparation, and sequencing

For Oxford Nanopore Technologies (ONT) sequencing, high-molecular weight (HMW) genomic DNA (gDNA) was isolated from an aliquot of a banked peripheral blood sample (stored at −80 °C after collection) of a colony-born adult female individual (Medusa, animal ID 6821) housed at the Duke Lemur Center (Durham, NC, USA), using the Qiagen MagAttract HMW DNA Kit (#67563; Qiagen, Hilden, Germany). A genomic sequencing library was prepared using the Oxford Nanopore Ligation Sequencing Kit (SQK-LSK110), sequenced on 2 Q20 PromethION flow cells (Oxford Nanopore Technologies, Oxford, UK), and base called using Guppy v.6.1.5 in the high accuracy setting, generating >3.7 million reads with an estimated N50 of 38.7 kb. The raw data was validated using fastQValidator version 0.1.1a (https://genome.sph.umich.edu/wiki/FastQValidator), and no errors were detected.

For Illumina sequencing, gDNA was extracted from an aliquot of the blood sample using the PureLink Genomic DNA Mini Kit and quantified using a Qubit 2.0 Fluorometer following the manufacturer’s instructions (Thermo Fisher Scientific, Waltham, MA, USA). Next, a sequencing library was prepared using the NEBNext Ultra II DNA PCR-free Library Prep Kit. In brief, gDNA was fragmented by acoustic shearing with a Covaris S220 instrument, cleaned up, and end-repaired. Adapters were ligated after adenylation of the 3′-ends. Prior to sequencing, DNA libraries were validated using a High Sensitivity D1000 ScreenTape on an Agilent TapeStation (Agilent Technologies, Palo Alto, CA, USA) and quantified using both a Qubit 4.0 Fluorometer and real-time PCR (Applied Biosystems, Carlsbad, CA, USA). The sequencing libraries were multiplexed and clustered onto a flow cell on an Illumina NovaSeq instrument and sequenced using a 2 × 150 bp paired-end configuration. Image analysis and base calling were conducted by the built-in NovaSeq Control Software. Raw sequence data (.bcl files) generated from Illumina NovaSeq was converted into .fastq files and de-multiplexed using Illumina's bcl2fastq v.2.20 software (allowing for 1 mismatch for index sequence identification), generating >850 million reads with a mean quality score of 38.9.

### Assembly

A high-quality aye-aye genome assembly was generated from a combination of ONT long reads and Illumina short reads and scaffolded using in situ Hi-C reads. Prior to the assembly, genome size, coverage, and repeat content were estimated based on the *k*-mer frequencies observed in the short-read data using Jellyfish v.2.3.0 (Marçais and Kingsford 2011) and GenomeScope v.2.0 (Ranallo-Benavidez *et al*. 2020). Following ONT’s best practices for primate-sized genomes (https://nanoporetech.com/resource-centre/human-genome-assembly-workflow), the long-read data were de novo assembled with Flye v.2.9.1 (Kolmogorov *et al*. 2019), using the recommended “*--–nano-hq*” flag for high-quality ONT reads together with the estimated genome size (“*--–genome-size*”), *i.e.* 2.4 Gb (Supplementary Fig. 1). To improve accuracy, the initial draft assembly was polished using 1 round of Racon v.1.4.20 (Vaser *et al*. 2017) together with the Illumina short-read sequencing data (with the “*–c*” flag enabled to trim adapter sequences), followed by 1 round of Medaka v.1.7.2 (https://github.com/nanoporetech/medaka) together with the ONT long-read sequencing data. Next, the polished assembly was scaffolded using the Juicer v.2.0 pipeline (Durand *et al*. 2016) together with genome-wide chromatin interaction data for the species, courtesy of the DNA Zoo Consortium (https://www.dnazoo.org/; Dudchenko *et al*. 2017). Within this framework, the assembly was first indexed using BWA *index* v.0.7.17 (Li and Durbin 2009), and Juicer’s built-in *generate_site_positions.py* script was used to identify MboI restriction enzyme cut sites within the indexed assembly. This indexed assembly and restriction enzyme information was then used together with the DNA Zoo Hi-C reads to create a list of chromatin interaction contact points. Using these contacts, 3D-DNA v.190716 (Dudchenko *et al*. 2017) was utilized to correct for potential mis-joins and generate a candidate scaffolded assembly. This candidate assembly was manually reviewed using Juicebox Assembly Tools v.2.17.00 (Dudchenko*et al*, in preprint) to create the final chromosome-level assembly. Lastly, the chromosome-level assembly was checked for contaminations using the NCBI Foreign Contamination Screen tool (https://github.com/ncbi/fcs). All software was executed using default settings.

### Quality assessment

The quality of the genome assembly was assessed using 3 criteria: contiguity, correctness, and completeness. First, the evaluation tool QUAST v.5.0.2 (Mikheenko *et al*. 2018) was used to measure assembly contiguity (N50 and L50). Second, Merqury v.1.3.0 (Rhie *et al*. 2020) was used, together with a *k*-mer database generated from the short-read data by Meryl v.1.4.1 (https://github.com/marbl/meryl), to assess *k*-mer completeness and correctness. Third, compleasm v.0.2.6 (Huang and Li 2023) was utilized to evaluate completeness based on the presence/absence of curated universal single-copy orthologous genes in the eukaryotic, mammalian, and primate libraries (eukaryota_odb10, mammalia_odb10, and primates_odb10, respectively; Manni *et al*. 2021). All software was executed using default settings.

### Annotation

#### Repeat annotation

Repeat families were identified by combining previous annotations with repeats detected de novo. In brief, previously identified repeats were first soft-masked in the final assembly using RepeatMasker v.4.1.5 (https://repeatmasker.org) based on the information obtained from the Lemuridae database in Dfam v.3.7 (Storer *et al*. 2021) and NCBI blastn (using the command “*-nolow -xsmall -gccalc -species Lemuridae -engine rmblast*” in the RepeatMasker compatible version RMBlast v.2.14.0; https://www.repeatmasker.org/rmblast/). Next, repeats were identified de novo using RECON v.1.08 (Bao and Eddy 2002), RepeatScout v.1.0.6 (Price *et al*. 2005), RMBlast v.2.14.1, and Tandem Repeats Finder v.4.09.1 (Benson 1999), embedded within RepeatModeler2 v.2.0.5 (Flynn *et al*. 2020). Lastly, annotated known and de novo repeats in the assembly were masked using RepeatMasker v.4.1.5 (with the following command line option: “*-xsmall -gccalc -lib consensi.fa.classified -engine rmblast*”). All software was executed using default settings.

#### Gene annotation

A 2-pronged gene annotation approach was implemented. First, BRAKER1 (Hoff *et al*. 2016) was used to generate ab initio gene predictions based on spliced alignments of RNA sequencing reads. Specifically, publicly available RNA sequencing data from a liver sample of an adult male individual previously housed at the Duke Lemur Center (Marvin, animal ID 6725) were downloaded from the functional genomics data collection (ArrayExpress accession number E-MTAB-4550; Berthelot *et al*. 2018) and mapped onto the final, repeat-masked assembly using STAR v.2.7.10b (Dobin *et al*. 2013). Second, due to the limited transcriptomic data available for the species, Liftoff v.1.6.3 (Shumate and Salzberg 2021) was used to project the human reference annotation release v.110 of the T2T-CHM13v2 assembly (GenBank accession number: GCA_009914755.4; Nurk *et al*. 2022) onto the DMad_hybrid assembly. In order to gain insights into gene predictions that may be specific to the aye-aye genome, tblastx embedded within BLAST+ v.2.12.0 (Camacho *et al*. 2009; Sayers *et al*. 2021) was then used to identify sequences unique to the ab initio gene predictions obtained from the RNA sequencing data. With these putatively aye-aye–specific gene models at hand, a blastn search was carried out against the Ensembl annotation release v.112 and resulting hits were analyzed in PANTHER v.18 (Thomas *et al*. 2022) to gather information about functional classifications. All software was executed using default settings.

#### Noncoding RNA and tRNA annotation

Noncoding RNAs were predicted using Infernal v.1.1.14 (Nawrocki and Eddy 2013) together with the information from the Rfam database v.14.10 (Kalvari *et al*. 2018, 2021). Transfer RNAs were predicted using tRNAscan-SE v.2.0.12 (Chan *et al*. 2021). All software was executed using default settings.

### Genome sequence comparison with other strepsirrhines

A genome sequence comparison was performed with 2 other strepsirrhine species for which high-quality genome assemblies are publicly available: the gray mouse lemur [*Microcebus murinus*; genome assembly: Mmur_3.0 (GenBank accession number: GCA_000165445.3); Larsen *et al*. 2017] and the ring-tailed lemur [*Lemur catta*; genome assembly: mLemCat1.pri (accession number: GCA_020740605.1); Palmada-Flores *et al*. 2022]. In brief, minimap2 v.2.22-r1101 (Li 2018, 2021) was used to generate whole-genome alignments between the final, repeat-masked aye-aye assembly and the gray mouse lemur and ring-tailed lemur assemblies, respectively. Whole-genome alignments were plotted using the asynt.R script (Kim *et al*. 2022) by filtering for alignments with syntenic block sizes of at least 20 kb. All software was executed using default settings.

## Results and discussion

**Fig. 1.**
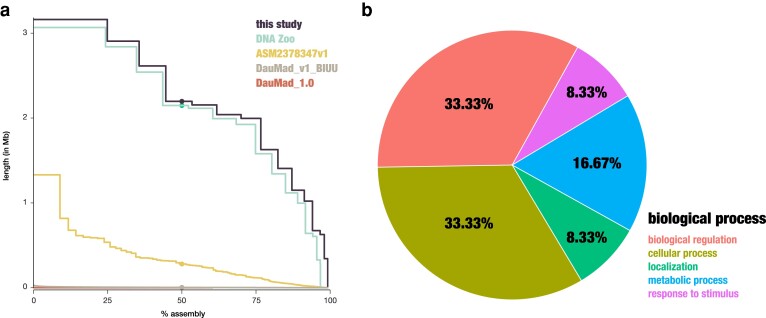
Aye-aye genome assembly. a) Improvements in contiguity (*x*-axis: percent of the genome within scaffolds/contigs; *y*-axis: scaffold/contig length in Mb) from the first aye-aye genome assemblies based on Illumina short-read data, DauMad_1.0 (Perry *et al*. 2012) and DauMad_v1_BIUU (Zoonomia Consortium 2020); to the short-read assembly scaffolded with genome-wide chromatin interaction data from DNA Zoo Consortium (https://www.dnazoo.org/); to the first long-read assembly, ASM2378347v1 (Shao *et al*. 2023); and to the hybrid assembly, DMad_hybrid, presented in this study. b) Functional classification of putatively aye-aye–specific gene models.

The genome of a female aye-aye (*D. madagascariensis*) housed at the Duke Lemur Center was de novo assembled using a combination of ONT long-read and Illumina short-read sequencing data and scaffolded using genome-wide chromatin interaction data. Briefly, using Oxford Nanopore sequencing, 82.69 Gb data with an estimated N50 of 38.7 kb (totaling a whole-genome coverage of >30×) were produced on 2 Q20 PromethION cells. Additionally, >850 million paired-end Illumina reads with a mean quality score of 38.9 were generated, corresponding to >100-fold genome-wide coverage. Long reads were de novo assembled using Flye (Kolmogorov *et al*. 2019) and polished using Racon (Vaser *et al*. 2017) and Medaka (https://github.com/nanoporetech/medaka), together with the high-quality short-read data to improve accuracy. The resulting 930 contigs (contig N50: 80 Mb) exhibit a total length of 2.44 Gb, similar to the length estimated from the raw genomic data (2.4 Gb) and within the range of previous assemblies for the species (2.41–2.86 Gb; Table 1). Compared to these older assemblies, the overall contiguity improved (Fig. 1a), with previous versions containing between ∼2,700 and >3.5 million contigs [in the long-read assembly, ASM2378347v1 (Shao *et al*. 2023), and in the short-read assembly, DauMad_1.0 (Perry *et al*. 2012), respectively), with N50 s ranging between 209 kb (DauMad_1.0) and 27 Mb (ASM2378347v1) and L50 s ranging from 193,839 (DauMad_1.0) to 5 (DNA Zoo Consortium). Contigs were scaffolded using Hi-C reads provided by the DNA Zoo Consortium (https://www.dnazoo.org/; Dudchenko *et al*. 2017) to produce a highly contiguous de novo assembly containing 696 scaffolds with an N50 of 215 Mb and a L50 of 5 (*k*-mer completeness: 98.27%). Taken together, compared to the previous assemblies, DMad_hybrid improved both the scaffold N50 [by 1,114-fold (DauMad_1.0), 567-fold (DauMad_v1_BIUU), 1-fold (DNA Zoo), and 8-fold (ASM2378347v1)] and contig N50 [by 383-fold (DauMad_1.0), 269-fold (DauMad_v1_BIUU), 372-fold (DNA Zoo), and 3-fold (ASM2378347v1)]. In agreement with earlier work reporting a diploid karyotype of 2*n* = 30 (Tattersall 1982), 15 chromosome-level scaffolds spanning the autosomes and chromosome X were identified that contained 99.17% of the assembly. Whole-genome alignments between these 15 chromosome-length aye-aye scaffolds, 33 gray mouse lemur (*M. murinus*) chromosomes (Larsen *et al*. 2017), and 29 ring-tailed lemur (*L. catta*) chromosomes (Palmada-Flores *et al*. 2022) revealed shared sequence homology between these strepsirrhine primates, despite their differences in karyotype (Supplementary Figs. 2 and 3, respectively).

Repetitive regions span a total of 35.02% of the aye-aye genome, with retroelements, DNA transposons, simple repeats, and low-complexity repeats representing 29.08%, 4.14%, 0.87%, and 0.20%, respectively. This repeat content is similar to that observed in other high-quality strepsirrhine genomes, with 29.38 and 39.91% repeat content in the gray mouse lemur and the ring-tailed lemur, respectively.

After masking repetitive regions, a 2-pronged approach was taken to annotate protein-coding regions in the species, based on information from spliced alignments of transcriptome data obtained from a liver tissue as well as external protein support from humans. Based on RNA sequencing data, 782 putatively aye-aye–specific gene models were identified that were enriched for cellular, metabolic, and regulatory processes (Fig. 1b); however, due to the limited transcriptomic data available for the species (i.e. a sample from a single individual and tissue type), this likely represents a biased view, and additional data will be required to obtain a more complete picture of changes unique to the aye-aye lineage. Overall, 18,858 protein-coding genes were identified in the *D. madagascariensis* assembly, similar to the total number of genes observed in the gray mouse lemur (20,671 genes) and in the ring-tailed lemur (19,990 genes). BUSCO analyses demonstrated that the aye-aye genome is near complete, containing 254 (99.61%), 9,220 (99.93%), and 13,668 (99.19%) highly conserved single-copy orthologous genes from the ortholog databases (odb10) of eukaryotes, mammals, and primates at the genome level (Table 1).

Finally, as genomic resources remain limited for strepsirrhine primates, this fully annotated, chromosome-level hybrid de novo assembly for the only extant member of the Daubentoniidae primate family presented here will open new avenues in primate comparative genomics in general and aye-aye conservation genetics specifically.

## Supplementary Material

jkae185_Supplementary_Data

## Data Availability

This whole-genome shotgun project has been deposited at DDBJ/ENA/GenBank under the accession JBFSEQ000000000. The version described in this paper is version JBFSEQ010000000. All sequence data has been deposited under NCBI BioProject PRJNA1085541. Supplemental material available at G3 online.
