# Supplementary material for: A hybrid genome assembly of the endangered aye-aye (Daubentonia madagascariensis)
Source: G3 (Bethesda). 2024 Aug 7;14(10):jkae185. doi: 10.1093/g3journal/jkae185 (PMC11457058; doi:10.1093/g3journal/jkae185)
Supplement: jkae185_Supplementary_Data [file jkae185_supplementary_data.pdf]

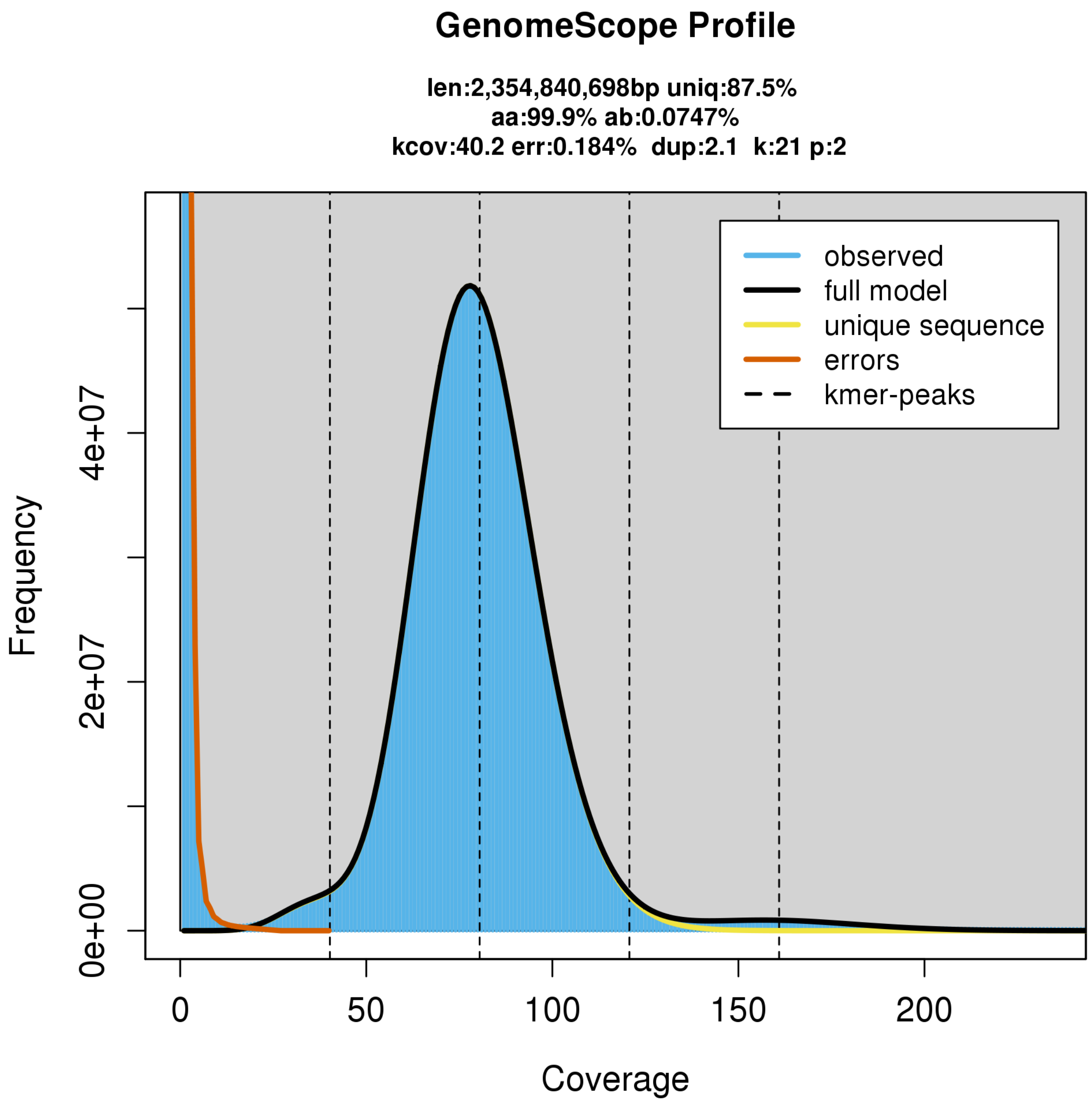

Figure S1. Genome size, coverage, and repeat content as estimated based on the *k*-mer frequencies observed in the short-read data.

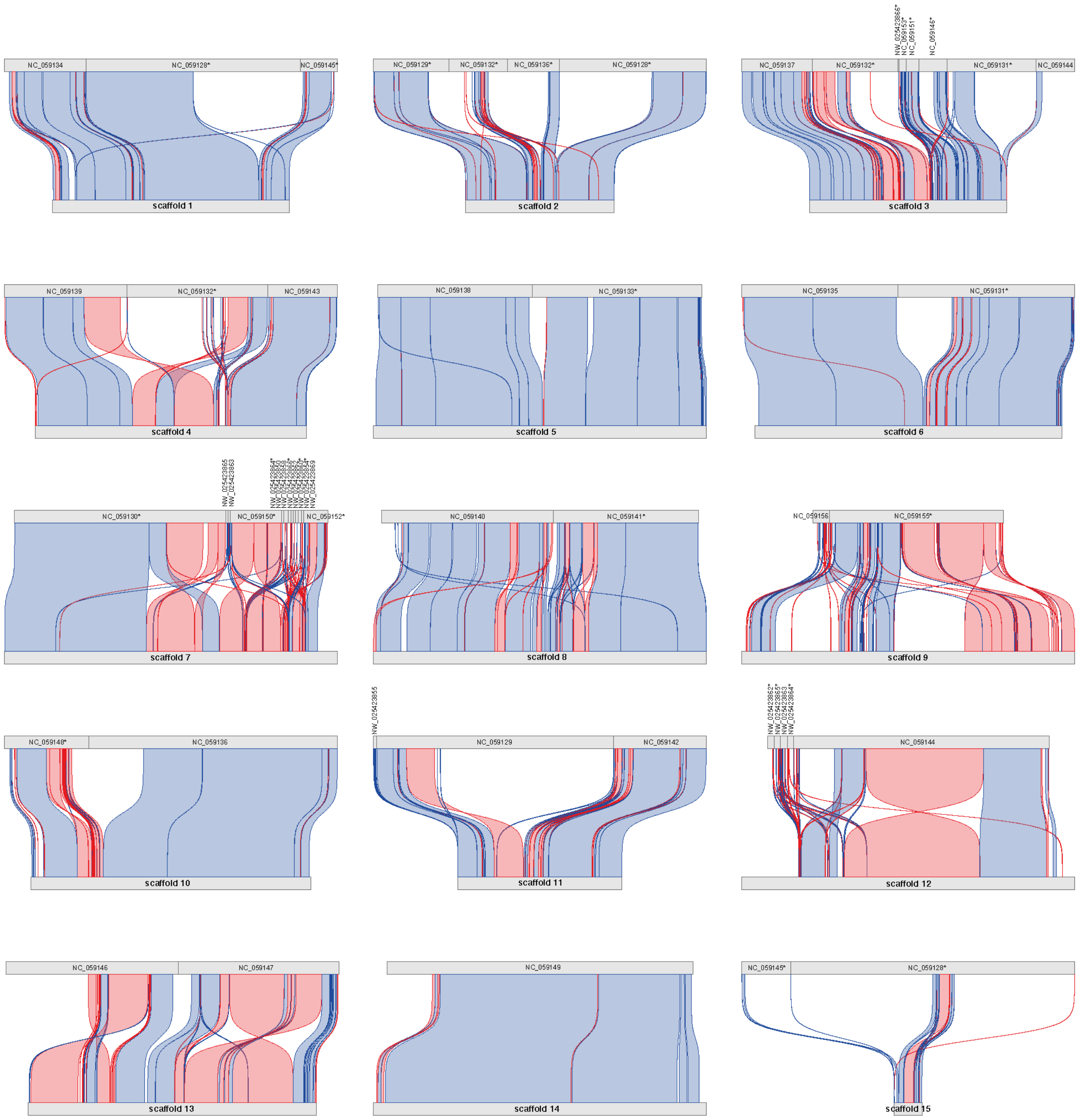

Figure S2. Whole genome alignments between the 15 chromosome-length aye-aye (*D. madagascariensis*) scaffolds (shown on the bottom of each panel) and the 33 gray mouse lemur (*Microcebus murinus*) chromosomes (top). Blue and red colors represent syntenic regions in the same and reverse orientation, respectively.

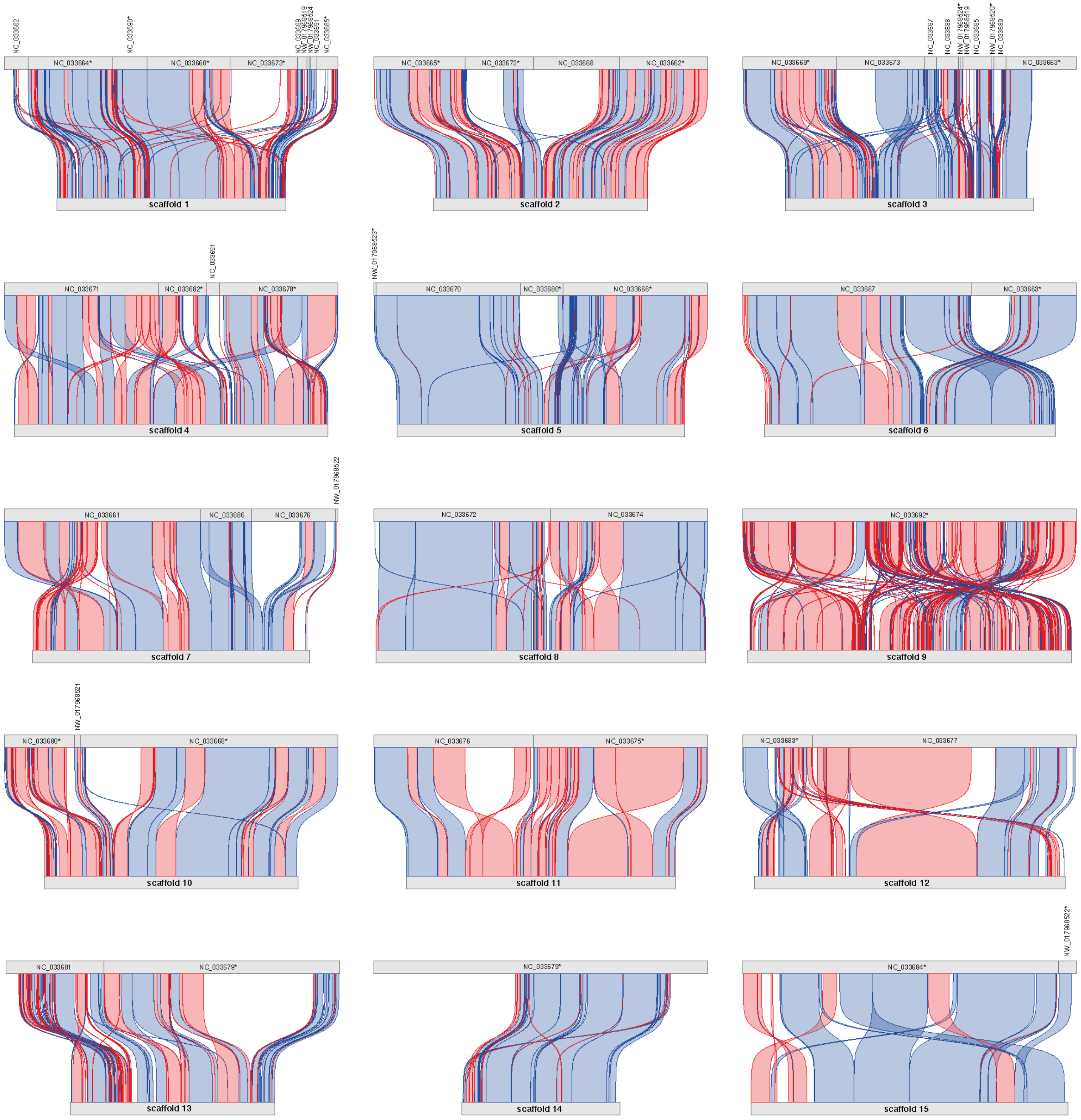

Figure S3. Whole genome alignments between the 15 chromosome-length aye-aye (*D. madagascariensis*) scaffolds (shown on the bottom of each panel) and the 29 ring-tailed lemur (*Lemur catta*) chromosomes (top). Blue and red colors represent syntenic regions in the same and reverse orientation, respectively.
